# Correlation of ultrasonography synovitis with disease activity and clinical response to etanercept treatment in juvenile idiopathic arthritis patients

**DOI:** 10.1590/1414-431X20198565

**Published:** 2019-11-21

**Authors:** Li Zhou, Xiaojie Gu

**Affiliations:** 1 Wuhan Children's Hospital, Wuhan Maternal and Child Healthcare Hospital, Tongji Medical College, Huazhong University of Science and Technology, Wuhan, China; 2 Department of Ultrasound, Children's Hospital of Nanjing Medical University, Nanjing, China

**Keywords:** Ultrasonography, Synovitis, Disease activity, Clinical response, Etanercept

## Abstract

This study aimed to investigate the correlation of ultrasonography (US) of synovitis with disease activity and clinical response to etanercept (ETN) in juvenile idiopathic arthritis (JIA) patients. Eighty-two JIA patients who underwent ETN treatment for 24 weeks were consecutively enrolled. US evaluations of 28 joints (shoulder, elbow, wrist, metacarpophalangeal, and proximal interphalangeal of hands and knee) at baseline were performed using grey-scale US and power doppler (PD) US, and US synovitis was defined as grey-scale abnormalities or PD abnormalities. Clinical response was assessed according to the ACRpedi 50 response criteria. In total, 2296 joints were scanned and 608 (26.5%) joints presented US synovitis, which was numerically higher than clinical synovitis (513 (22.3%)). The mean number of joints showing synovitis on US was 7.42±3.35, which was also numerically higher than that of clinical synovitis (6.26±2.70). The number of joints showing synovitis on US was positively correlated with C-reactive protein, erythrocyte sedimentation rate, number of joints with active disease, number of joints with limited range of motion, physician's global assessment of disease activity, parent/patient global assessment of overall well-being, and childhood health assessment questionnaire score. Most interestingly, the baseline number of joints showing synovitis on US was increased in ACRpedi 50 response JIA patients compared to non-response JIA patients, and it serves as an independent predictive factor for higher clinical response to ETN treatment. In conclusion, US is a more sensitive test to evaluate subclinical synovitis and disease activity in JIA patients, and US synovitis might serve as a marker for predicting increased clinical response rate to ETN treatment.

## Introduction

Juvenile idiopathic arthritis (JIA), as a chronically inflammatory and autoimmune disease, is a critical health issue affecting children and adolescents worldwide (1,2). The incidence and prevalence of JIA vary greatly among diverse areas and populations, and a recent systematic review reports that the JIA incidence ranges from 1.6 to 23 per 100,000 persons and prevalence ranges from 3.8 to 400 per 100,000 persons (3). The precise etiology of JIA is still largely unknown, while it is considered that the interaction between environmental factors and multiple genes/pathways is the most relevant working mechanism for the development of JIA (4). Although great treatment improvements have been achieved with imaging technology progress, novel drugs application (especially TNF-α inhibitors such as etanercept (ETN)), and efficient treating strategies, there are still a proportion of JIA patients that fail to respond or relapse in a short time (5,6). Therefore, exploration of potential markers for disease monitoring and treatment efficacy prediction is essential to further improve outcomes of JIA patients.

Ultrasonography (US), initially applied and recommended for evaluation of subclinical synovitis, disease activity, and treatment response in rheumatoid arthritis, has been introduced for JIA examination recently (7,8). US presents several advantages compared to other imaging technologies such as easy accessibility, fast imaging, dynamic feature, no exposure to radiation, and sedation-free (9). Due to the above-mentioned benefits, US was proposed to be applied in JIA disease monitoring and management as a feasible imaging technology, especially for detection of synovitis (10). However, few studies have assessed the correlation of US synovitis with comprehensive clinical disease activity of JIA, and no study has reported the predictive value of US synovitis for treatment response to ETN. Therefore, the present study detected US synovitis of 28 joints by greyscale and power doppler (PD) US in 82 JIA patients who underwent ETN treatment, and aimed to investigate the correlation of US synovitis with disease activity and clinical response to ETN.

## Material and Methods

### Participants

Eighty-two JIA patients from the Children's Hospital of Nanjing Medical University were consecutively enrolled in this study from January 2015 to November 2017. The inclusion criteria were: 1) diagnosed as JIA according to the International League of Associations for Rheumatology criteria (11); 2) about to receive ETN treatment; and 3) able to be followed-up regularly. This study was conducted in line with the Declaration of Helsinki and was approved by the Ethics Committee of Children's Hospital of Nanjing Medical University. All the patients or their parents (guardians) provided written informed consents.

### Data collection

After enrollment, patients' characteristics were recorded including: 1) demographic features: age, gender, height, weight, and disease duration; 2) JIA subtypes: oligoarthritis, rheumatoid factor (RF) negative-polyarthritis, RF-positive polyarthritis, systemic, enthesitis-related arthritis, or psoriatic; 3) clinical features: C-reactive protein (CRP), erythrocyte sedimentation rate (ESR), joints with active arthritis, joints with limited range of motion, physician's global assessment of disease activity, parent/patient global assessment of overall well-being, and childhood health assessment questionnaire (CHAQ) scores; 4) initiation of treatment: ETN, methotrexate (MTX), leflunomide (LEF), and other disease-modifying antirheumatic drugs (DMARDs).

### US synovitis assessments at baseline

US evaluation of 28 joints (shoulder, elbow, wrist, metacarpophalangeal (MCP), proximal interphalangeal of hands (PIP), and knee) at baseline were performed for evaluation of the disease and its potential to predict response to ETN in all patients (n=82). US was done with MyLab 70 (Biosound Esaote, USA) with a 6–18 MHz linear probe and a Logiq S8 (GE, USA) with an 8–18 MHz linear probe according to the settings described in a previous study (12), which were freely provided for this study. Because US was not provided for free after treatment for 24 weeks due to lack of sufficient funds, only a proportion of patients (n=39) underwent US detection after 24 weeks. US synovitis was defined as a joint that presented with greyscale abnormalities (joint effusion and hypertrophied synovium) or PD abnormalities according to several reports (7,12). The number of joints showing synovitis on US were calculated for further analysis.

### Treatment

All 82 JIA patients received ETN treatment for 24 weeks as subcutaneous injections, twice weekly, at the dose of 0.4 mg/kg. According to the disease conditions, MTX, LEF, or other DMARDs were combined.

### ACRpedi 50 response assessment

After the 24-week treatment, clinical response was assessed according to the ACRpedi 50 response criteria: 50% improvement from baseline in at least 3 of any 6 variables of the JIA core sets, with no more than 1 variable worsening by more than 30% (13). The 6 variables were ESR, joints with active arthritis, joints with limited range of motion, physician's global assessment of disease activity, parent/patient global assessment of overall well-being, and CHAQ.

### Statistical analysis

Statistical analysis was performed using SPSS 22.0 software (IBM, USA). Data are reported as means±SD or count (percentage). Comparison between two groups was done by *t*-test, correlation between two variables was assessed by Pearson’s test, and factors affecting clinical response were assessed by univariate and multivariate logistic regression analysis. A P<0.05 was considered significant.

## Results

### Patients' characteristics

The mean age of the enrolled 82 JIA patients was 6.8±2.8 years with 27 males and 55 females. The disease duration was 2.78±1.74 years. There were 25 (31%) oligoarthritis, 25 (31%) RF-negative polyarthritis, 12 (15%) RF-positive polyarthritis, 10 (12%) systemic arthritis, 8 (10%) enthesitis-related arthritis, and 2 (2%) psoriatic JIA subtypes. The mean CRP and ESR were 38.5±23.0 mg/L and 32.7±20.0 mm/h, respectively. The other detailed characteristics of JIA patients are listed in Table 1.

**Table 1.** Patient characteristics.

Parameters	JIA patients (n=82)
Demographic features	
Age (years)	6.8±2.8
Gender (male/female)	27/55
Height (cm)	119±18
Weight (kg)	24.1±8.5
Disease duration (years)	2.78±1.74
JIA Subtype (n/%)	
Oligoarthritis	25 (31)
RF negative polyarthritis	25 (31)
RF positive polyarthritis	12 (15)
Systemic	10 (12)
Enthesitis-related arthritis	8 (10)
Psoriatic	2 (2)
Clinical features	
CRP (mg/L)	38.5±23.0
ESR (mm/h)	32.7±20.0
Joints with active arthritis (n)	6.6±3.1
Joints with limited range of motion (n)	3.8±2.0
Physician's global assessment of disease activity	5.9±1.7
Parent/patient global assessment of overall well-being	5.5±2.0
CHAQ	1.7±0.6
Treatments (n/%)	
ETN	82 (100)
MTX	20 (24)
LEF	39 (48)
Other DMARDs	16 (20)

Data are reported as means±SD or count (percentage). JIA, juvenile idiopathic arthritis; RF: rheumatoid factor; CRP: C-reactive protein; ESR: erythrocyte sedimentation rate; CHAQ: Childhood Health Assessment Questionnaire; ETN: etanercept; MTX: methotrexate; LEF: leflunomide; DMARDs: disease-modifying antirheumatic drugs.

### Presence of US synovitis

The detection rate of US synovitis was numerically higher compared to the detection rate of clinical synovitis regarding shoulder, elbow, wrist, MCP, PIP, and knee joints at baseline; in a total of 2296 scanned joints, 608 (26.5%) joints presented US synovitis, which was numerically higher compared to 513 (22.3%) joints with clinical synovitis (Table 2). Also, the mean number of joints showing synovitis on US was 7.42±3.35, which was numerically higher than the mean number of joints showing clinical synovitis (6.26±2.70) at baseline (Table 3).

**Table 2.** Number of baseline ultrasonography (US) abnormalities and clinical synovitis in 28 joints.

Items	n	Clinical synovitis	Greyscale abnormalities	PD abnormalities	US synovitis
Shoulder	164	19 (11.6)	24 (14.6)	11 (6.7)	24 (14.6)
Elbow	164	35 (21.3)	42 (25.6)	18 (11.0)	42 (25.6)
Wrist	164	82 (50.0)	88 (53.7)	37 (22.6)	88 (53.7)
MCP	820	146 (17.8)	188 (22.9)	58 (7.1)	188 (22.9)
PIP	820	110 (13.4)	139 (17.0)	51 (6.2)	139 (17.0)
Knee	164	121 (73.8)	127 (77.4)	75 (45.7)	127 (77.4)
Scanned joints	2296	513 (22.3)	608 (26.5)	250 (10.9)	608 (26.5)

Data are reported as count and percentage. PD: power doppler; MCP: metacarpophalangeal; PIP: proximal interphalangeal of hands.

**Table 3.** Number of joints showing synovitis on ultrasonography (US) and clinical synovitis.

Parameters	JIA patients (n=82)
Number of joints showing clinical synovitis	6.26±2.70
Number of joints showing greyscale abnormalities	7.42±3.35
Number of joints showing PD abnormalities	3.05±1.86
Number of joints showing synovitis on US	7.42±3.35

Data are reported as means±SD. JIA: juvenile idiopathic arthritis; PD: power doppler.

### Correlation of US synovitis with patients' features

**Figure 1.**
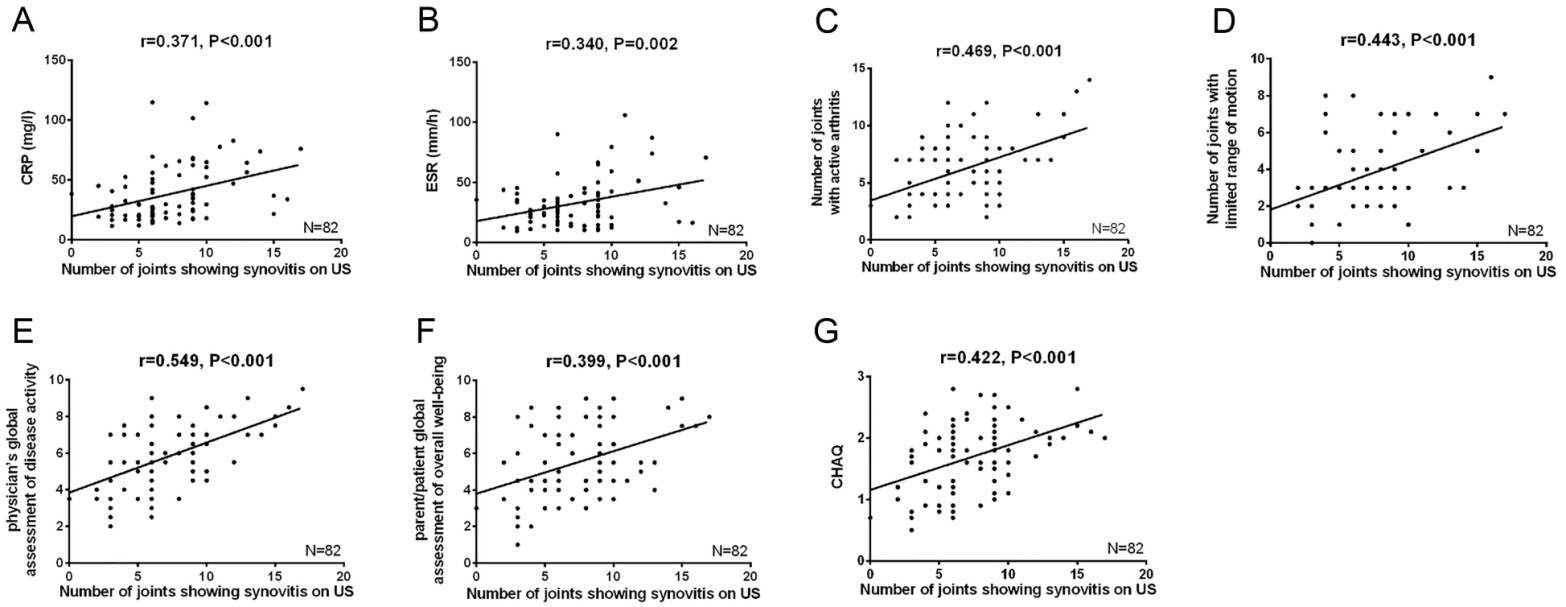
Correlation between ultrasonography (US) synovitis with patients' features. Number of joints showing synovitis on US positively associated with CRP (**A**), ESR (**B**), number of joints with active disease (**C**), number of joints with limited range of motion (**D**), physician's global assessment of disease activity (**E**), parent/patient global assessment of overall well-being (**F**), and childhood health assessment questionnaire (CHAQ) score (**G**). Correlation was detected using Pearson’s test and comparison between two groups was detected using *t*-test. P<0.05 was considered as significant. CRP, C-reactive protein; ESR, erythrocyte sedimentation rate.

The number of joints showing synovitis on US at baseline was positively correlated with CRP (P<0.001, Figure 1A), ESR (P=0.002, Figure 1B), number of joints with active disease (P<0.001, Figure 1C), number of joints with limited range of motion (P<0.001, Figure 1D), physician's global assessment of disease activity (P<0.001, Figure 1E), parent/patient global assessment of overall well-being (P<0.001, Figure F), and CHAQ score (P<0.001, Figure 1G). However, no correlation with age (P=0.929), gender (P=0.204), height (P=0.874), weight (P=0.806), or disease duration (P=0.664) was observed. These data indicated US synovitis correlated with increased disease activity in JIA patients.

### Correlation of US synovitis with clinical response

**Figure 2.**
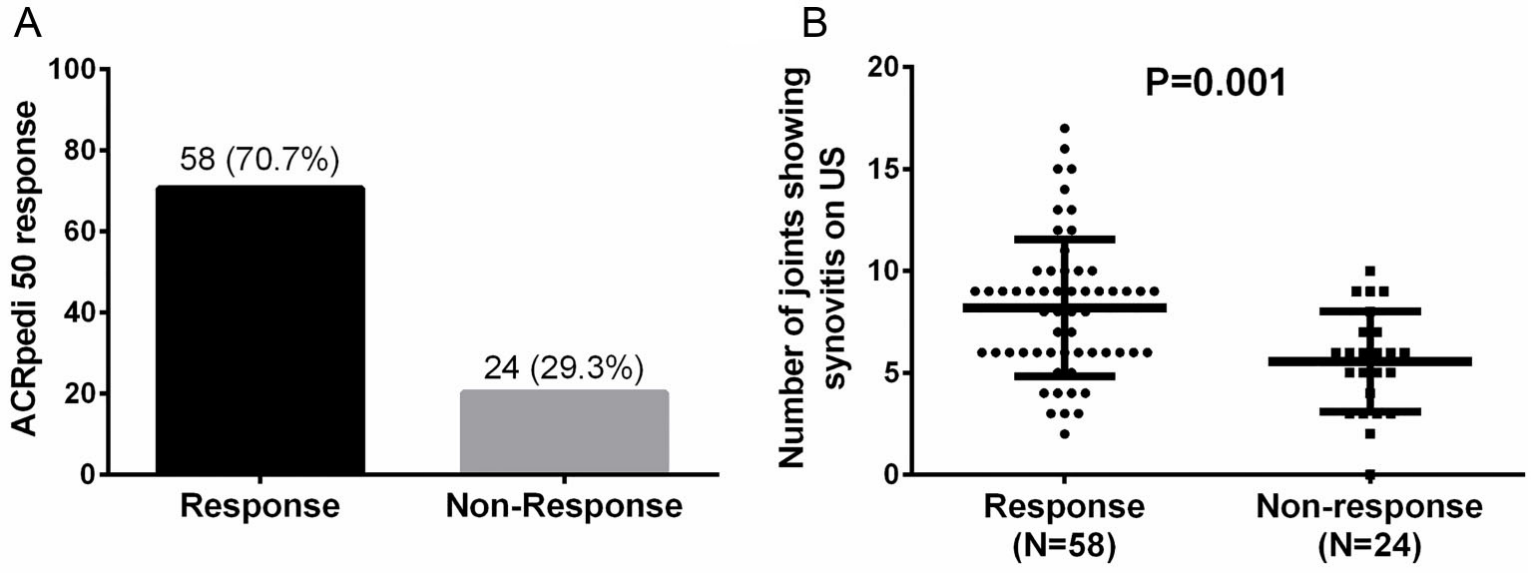
Ultrasonography (US) synovitis in ACRpedi 50 response and non-response patients. A total of 58 patients achieved ACRpedi 50 response to etanercept at 24 weeks, with a response rate of 70.7%, **A**, Number of joints showing synovitis on US at baseline was higher in response patients than non-response patients. **B**, Comparison between two groups was detected using *t*-test. P<0.05 was considered to be significant.

After the 24-week ETN treatment, 58 (70.7%) patients achieved ACRpedi 50 response while 24 (29.3%) patients failed (Figure 2A). The number of joints showing synovitis on US at baseline was greater in response patients compared to non-response patients (P=0.001, Figure 2B). This suggested US synovitis had potential to predict clinical response to ETN.

### Factors predicting clinical response to ETN treatment

Univariate logistic regression model revealed that the number of joints showing synovitis on US at baseline (P=0.001, OR=1.489, 95%CI: 1.189–1.865) and CRP (P=0.003, OR=1.064, 95%CI: 1.021–1.108) predicted higher ACRpedi 50 response, while systemic JIA subtype (P=0.032, OR=0.222, 95%CI: 0.056–0.877) predicted lower ACRpedi 50 response (Table 4). Only factors with P values below 0.1 in univariate logistic regression were further analyzed by multivariate logistic regression analysis (Table 5), which showed that the number of joints with synovitis on US at baseline (P=0.010, OR=1.438, 95%CI: 1.091–1.897) and CRP (P=0.047, OR=1.050, 95%CI: 1.001–1.102) were independent predictive factors for higher ACRpedi 50 response, while systemic JIA subtype (P=0.033, OR=0.152, 95%CI: 0.027–0.856) was an independent predictive factor for lower ACRpedi 50 response. These data implied US synovitis served as marker for predicting higher clinical response rate to ETN treatment.

**Table 4.** Factors predicting ACRpedi 50 response to etanercept treatment by univariate logistic regression model.

Factors	Univariate logistic regression			
	P value	OR	95% CI	
			Lower	Higher
Number of joints showing synovitis on US at baseline	0.001	1.489	1.189	1.865
Age	0.641	0.960	0.807	1.141
Gender (male)	0.642	1.278	0.455	3.592
Height	0.927	0.999	0.972	1.026
Weight	0.408	0.977	0.925	1.032
Disease duration	0.549	0.920	0.700	1.209
Oligoarthritis	0.489	1.462	0.499	4.280
R-negative polyarthritis	0.719	0.829	0.299	2.299
RF-positive polyarthritis	0.738	0.800	0.216	2.957
Systemic	0.032	0.222	0.056	0.877
Enthesitis-related arthritis	–	–	–	–
Psoriatic	–	–	–	–
CRP	0.003	1.064	1.021	1.108
ESR	0.625	1.006	0.981	1.032
Joints with active arthritis	0.061	1.214	0.991	1.486
Joints with limited range of motion	0.397	1.113	0.869	1.426
Physician's global assessment of disease activity	0.289	1.170	0.875	1.564
Parent/patient global assessment of overall well-being	0.460	1.098	0.857	1.407
CHAQ	0.505	1.326	0.579	3.038
MTX	0.300	1.905	0.563	6.440
LEF	0.442	0.688	0.264	1.787
Other DMARDs	0.309	2.022	0.520	7.864

Enthesitis-related arthritis and psoriatic could not be analyzed due to lack of events. P<0.05 was considered significant. US: ultrasonography; RF: rheumatoid factor; CRP: C-reactive protein; ESR: erythrocyte sedimentation rate; CHAQ: childhood health assessment questionnaire; MTX: methotrexate; LEF: leflunomide; DMARDs: disease-modifying antirheumatic drugs.

**Table 5.** Factors predicting ACRpedi 50 response to etanercept treatment by multivariate logistic regression model.

Factors	Multivariate logistic regression			
	P value	OR	95% CI	
			Lower	Higher
Number of joints showing synovitis on US at baseline	0.010	1.438	1.091	1.897
Systemic	0.033	0.152	0.027	0.856
CRP	0.047	1.050	1.001	1.102
Joints with active arthritis	0.383	1.118	0.870	1.435

Factors with P<0.1 in univariate logistic regression were further analyzed by multivariate logistic regression analysis. P<0.05 was considered significant. US: ultrasonography; CRP: C-reactive protein.

### Attenuation of US synovitis after 24-week treatment

In the 39 patients who underwent US detection after 24-week ETN treatment, the number of joints showing synovitis on US was greatly reduced (4.01±1.68) compared to baseline (7.29±3.14) (P<0.001). Also, mean number of joints showing clinical synovitis was also decreased at 24 weeks (2.85±1.53) compared to baseline (6.14±2.15) (P<0.001).

## Discussion

In the current study, we found that in JIA patients who underwent ENT treatment: 1) US synovitis occurrence was 26.5%, and the detection rate was higher compared to clinical synovitis; 2) US synovitis was correlated with increased disease activity; 3) US synovitis predicted higher clinical response to ETN treatment.

The detection and management of synovitis, as a chief factor responsible for inflammation and structural joint damage in JIA, has attracted great attention (14). Traditionally, synovitis is detected clinically by assessing active, tender, or swollen joints, and several systemic or targeted anti-inflammation treatments are applied (15,16). Along with the wide utilization of US imaging in several chronic arthritis diseases such as osteoarthritis and rheumatoid arthritis (8,17), its application has been gradually introduced to JIA management (14). Several reports disclose that US-detected synovitis rate is higher compared to clinically-assessed synovitis rate. For instance, a study examined 1660 joints in 32 JIA patients clinically and by US imaging, and revealed that 10% of joints presented with US synovitis while only 6.3% showed clinical synovitis (16); another study assessed 1120 joints in 40 JIA patients, and found an 18.8% US synovitis rate while the clinical synovitis rate was 17.5% (14). In our study, we also observed that detection rate of US synovitis was higher compared to detection rate of clinical synovitis, which was in line with previous studies. The occurrences of US synovitis (26.5%) and clinical synovitis (22.3%) in our study were both numerically elevated compared with previous studies, which might be due to patients in this study presenting more severe disease conditions who were about to receive ETN treatment, thus they showed an increased number of joints with synovitis.

US synovitis also has potential to be used as a disease-monitoring marker in arthritis disease. For instance, a study detected US synovitis of wrist joints in 50 early rheumatoid arthritis patients, and disclosed PD-US synovitis showed positive correlation with TNF-α, IL-6, and angiopoietin-1 and -2 levels (18). Another study used a 7-joint US synovitis score in 50 RA patients, and found that the scores were greatly correlated with disease activity assessed by 3 different composite disease indices (19). Few studies report the application of US synovitis as disease-activity marker in JIA. A previous study assessed 28 joints for US abnormalities in 40 JIA patients, and found that US synovitis scores (including synovial hyperplasia, joints effusion score, and PD-US score) correlated positively with pain VAS score, swollen joints count, tender joints count, ESR, CRP, and disease activity score-28 joints (DAS28)-CRP (14). Another study detected clinically active joints by PD-US in 32 JIA patients, and observed that PD-US synovitis is more sensitive than ESR or CRP in identification of active disease (20). In our study, we observed that the number of joints showing synovitis on US at baseline was positively correlated with comprehensive disease activity indexes including CRP, ESR, number of joints with active disease, number of joints with limited range of motion, physician's global assessment of disease activity, parent/patient global assessment of overall well-being, and CHAQ score. These data might be due to: 1) more joints with US synovitis promoted elevated inflammation and immune responses, thus led to higher disease activity; 2) more joints with US synovitis increased inflammation, pain, and action limits, thus resulted in worse disease activity score and quality of life.

Apart from the above-mentioned application of US in synovitis, it may serve as a potential marker for disease prognosis as well in several types of arthritis. A study with 141 rheumatoid arthritis (RA) patients who underwent synthetic and biologic DMARDs treatments for 12 months underwent wrist, MCP 2/3, PIP 2/3, and metatarsophalangeal 2/5 gray scale US and PD-US found that lower DAS28 score and PD-US score correlated with higher clinical remission after treatment (21). Another study with 151 RA patients who were treated with single conventional synthetic DMARD or a biologic DMARD agent for 4 months had 24 joints evaluated by US. The authors observed that a higher total US score predicted increased treatment response in these patients (22). The discrepancy of the above data may be the result of remission being defined as an absolute/specific value, thus a baseline lower disease activity (for instance, lower PDUS score) would lead to higher possibility of remission afterward (21). Because treatment response is defined as the change in disease activity, higher disease severity at baseline might have more opportunity for the indexes to decline to the standard-of-treatment response (22). As for JIA, no study revealed the predictive value of US abnormities for clinical response to ETN until now; only some studies report that the presence of US synovitis predicts disease flare in inactive JIA patients (12,23).

In the present study, we found that the baseline number of joints showing synovitis on US was correlated with increased response in JIA patients, serving as an independent predictive factor for higher clinical response rate to ETN treatment. The possible explanations are: 1) increased number of joints showing synovitis on US correlates with higher disease inflammation and activity, and ETN is a target anti-inflammation agent, thus ETN presented better efficacy in these high-inflammation patients; 2) clinical treatment in our study was evaluated using ACRpedi 50 response criteria (13), which used the change of disease severity indexes instead of direct disease severity indexes as measurements, thus higher baseline disease severity indexes (increased number of US synovitis) had more space to decline, thus the increased number of US synovitis correlated with higher clinical response to ETN.

Several limitations are present in this study: 1) due to patients' unwillingness, US examinations after treatments were only done in a few patients, thus the effect of ETN on US synovitis attenuation was not analyzed in our study; 2) the sample size of this study was relatively small because of the high cost of ETN; 3) most patients in this study came from Middle China, thus multiple-region and multiple-center validation is needed in the future; 4) a commonly affected joint, the ankle, was not included in the US evaluation in this study, which should be further explored in the future.

In conclusion, US is a more sensitive test to evaluate subclinical synovitis and disease activity in JIA patients, and US synovitis might serve as a marker for predicting increased clinical response rate to ETN treatment.
